# Multi-level, multi-scale resource selection functions and resistance surfaces for conservation planning: Pumas as a case study

**DOI:** 10.1371/journal.pone.0179570

**Published:** 2017-06-13

**Authors:** Katherine A. Zeller, T. Winston Vickers, Holly B. Ernest, Walter M. Boyce

**Affiliations:** 1 Department of Environmental Conservation, University of Massachusetts, Amherst, Massachusetts, United States of America; 2 Karen C. Drayer Wildlife Health Center, School of Veterinary Medicine, University of California Davis, Davis, California, United States of America; 3 Department of Veterinary Sciences, University of Wyoming, Laramie, Wyoming, United States of America; University of Alberta, CANADA

## Abstract

The importance of examining multiple hierarchical levels when modeling resource use for wildlife has been acknowledged for decades. Multi-level resource selection functions have recently been promoted as a method to synthesize resource use across nested organizational levels into a single predictive surface. Analyzing multiple scales of selection within each hierarchical level further strengthens multi-level resource selection functions. We extend this multi-level, multi-scale framework to modeling resistance for wildlife by combining multi-scale resistance surfaces from two data types, genetic and movement. Resistance estimation has typically been conducted with one of these data types, or compared between the two. However, we contend it is not an either/or issue and that resistance may be better-modeled using a combination of resistance surfaces that represent processes at different hierarchical levels. Resistance surfaces estimated from genetic data characterize temporally broad-scale dispersal and successful breeding over generations, whereas resistance surfaces estimated from movement data represent fine-scale travel and contextualized movement decisions. We used telemetry and genetic data from a long-term study on pumas (*Puma concolor*) in a highly developed landscape in southern California to develop a multi-level, multi-scale resource selection function and a multi-level, multi-scale resistance surface. We used these multi-level, multi-scale surfaces to identify resource use patches and resistant kernel corridors. Across levels, we found puma avoided urban, agricultural areas, and roads and preferred riparian areas and more rugged terrain. For other landscape features, selection differed among levels, as did the scales of selection for each feature. With these results, we developed a conservation plan for one of the most isolated puma populations in the U.S. Our approach captured a wide spectrum of ecological relationships for a population, resulted in effective conservation planning, and can be readily applied to other wildlife species.

## Introduction

Almost 40 years ago, Johnson [1] established a hierarchical framework for examining wildlife habitat use. He proposed ordering habitat selection from Level I (i.e. selection of the geographical range of a species) to Level IV (i.e. selection of feeding sites) and argued that, “This hierarchy of selection has a unifying nature. Habitat usage studies and investigations of feeding are no longer qualitatively distinct; they are simply of different orders”. Johnson’s hierarchical framework set the stage for much of the recent research, thinking, and understanding of how wildlife use habitat [2].

Examining selection at these different hierarchical levels encourages wildlife habitat use to be understood as a cascading process conditional upon higher levels of selection. For example, avoidance of human development may be weak at one level of selection but strong at another [3–5]. Instead of using the outputs from each hierarchical level independently, DeCesare et al. [4] proposed integrating the predictive surfaces from resource selection functions (RSFs; [6]), across levels of selection. DeCesare et al. [4] referred to these integrated predictive surfaces as Scale Integrated RSFs. Following the nomenclature of McGarigal et al. [2] we will hereafter refer to these as Multi-level RSFs (ML-RSFs). These ML-RSFs provide the relative probability of habitat use at a lower level, for example Level III, conditional upon the relative probability of habitat use at a higher level, for example Level II.

In addition to being conceptually appealing, ML-RSFs offer a number of advantages. First, they may reveal differences in selection at different hierarchical levels, which is useful for conservation and management of wildlife. Second, they provide a single surface that synthesizes habitat use across different levels, making it easier for managers to digest and use [4]. Third, they are relatively simple to derive since the hierarchical conditional probabilities collapse into a simple equation that is the product of the two relative probability surfaces. Studies that are unable to use the same data for each hierarchical level, but produce RSFs for different levels of selection and obtain their product, are not truly conditionally nested, but are conceptually similar and approximate hierarchically nested levels (e.g. [5–7]).

We expand the concept of ML-RSFs to resistance surfaces. Resistance surfaces quantify how environmental parameters affect wildlife movement and are the basis for most connectivity and corridor models [8]. Resistance surfaces are conceptualized as the union of three processes, (1) the willingness of an organism to cross a particular landscape feature, (2) the physiological cost of moving across a landscape feature, and (3) the reduction in survival due to crossing a landscape feature [8, 9]. However, in practice, it is difficult to estimate resistance for these three processes simultaneously. Typically, one of two data types are promoted as the gold standards for estimating resistance for wildlife: movement data or genetic data [10–13]. Movement data have been promoted as an effective way to estimate resistance at fine scales, both temporally and spatially, through context-dependent analyses of animal pathways [10, 13, 14]. Resistance surfaces from movement data approximate the first resistance concept, the willingness of an individual to cross a feature. Genetic data, as used in landscape genetic analyses, estimate resistance by correlating environmental variables with estimates of gene flow. Through this process, variables that minimize fitness costs, increase survival, and result in successful dispersal and breeding are identified [12]. Thus, resistance surfaces derived from genetic data approximate the second two resistance processes, physiological and survival costs.

Previous studies have attempted to determine if movement data can be used to predict genetic connectivity, and vice versa [10, 15]. To date, the results have been equivocal. Cushman and Lewis [10] found many similarities between the resistance surfaces derived from these two data types for black bears in Idaho. Resistance surfaces between genetic data and an early season movement model had a correlation coefficient of ~0.4 and shared the same variables with the same relationship to movement. However, correlation between the genetic resistance surface and the resistance surface derived from the late season movement model was much lower at ~0.1. In other analyses on roe deer in France, Coulon et al. [15, 16] found an avoidance of woodland with movement data and a preference for woodland with genetic data.

Instead of trying to compare resistance surfaces derived from these two data types, we argue their best use may be by combing them into a multi-level resistance surface (ML-RS), akin to the ML-RSFs described above. Genetic data reflect movement, dispersal and breeding over generations which is analogous to a higher organizational level that represents broader scales and longer temporal windows. Pathway data, on the other hand, represents movement over much shorter temporal periods and reflect real-time movement decisions by individuals, which is analogous to a lower organizational level. Just as it is difficult to discern Level III habitat selection by using a home range selection function, so too is it difficult to discern fine-scaled movement patterns with a landscape genetic analysis. Conversely, it is difficult to identify variables that result in successful dispersal and breeding using only movement data. By integrating these surfaces, all three resistance processes can be estimated.

Analyzing species habitat use and movement across hierarchical levels and integrating the results captures selection and behavioral processes at different extents and results in stronger inference and predictive abilities than using a single level alone [2, 4, 6, 17]. Multi-level selection and movement models can be improved upon conducting multi-scale analyses within each hierarchical level. Not only do individuals select for environmental variables at different scales within a hierarchical level [18–21], individuals may also select different scales for the same environmental variable among hierarchical levels [5, 7]. These scales of selection are most often represented by summarizing each variable of interest with ecological neighborhoods of varying sizes [22]. The scale that produces the highest model performance for each variable is often referred to as the ‘characteristic scale’ of selection [23]. For example, in our previous work, we have shown that in Level III selection, pumas respond to human development variables at coarse scales and to natural features at finer scales [21]. Bauder et al. [5] found different scales of selection for multiple environmental variables for eastern indigo snakes across Levels II and III selection. Developing multi-level, multi-scale models captures a wide spectrum of ecological relationships for a population resulting in more meaningful conservation plans.

As a case study for developing a multi-level, multi-scale conservation plan, we use data from a long-term study on pumas (*Puma concolor*) in a highly developed region of southern California. Over 20 years ago, Beier [24, 25] concluded that the coastal Santa Ana Mountains puma population was becoming isolated due to increased urbanization and long-term population viability was dependent on maintaining permeability to the eastern Peninsular Ranges. More recent genetic analyses have shown that the Santa Ana Mountains population not only has the lowest genetic diversity of any population in California, but also shows signs of inbreeding [26], indicating a heightened need for connectivity. This is complicated by low adult survival rates in the Santa Ana Mountains population (55.8%), with humans (mostly via vehicle strikes) causing the majority of puma deaths [27]. A detailed puma conservation plan accounting for connectivity between the Santa Ana Mountains and the eastern Peninsular Ranges is needed and will directly inform conservation preserve design in northern San Diego County. We present a multi-level, multi-scale approach to conservation planning for pumas in northern San Diego and southern Riverside and Orange Counties.

## Materials and methods

### Study area and GPS collar data

**Fig 1:**
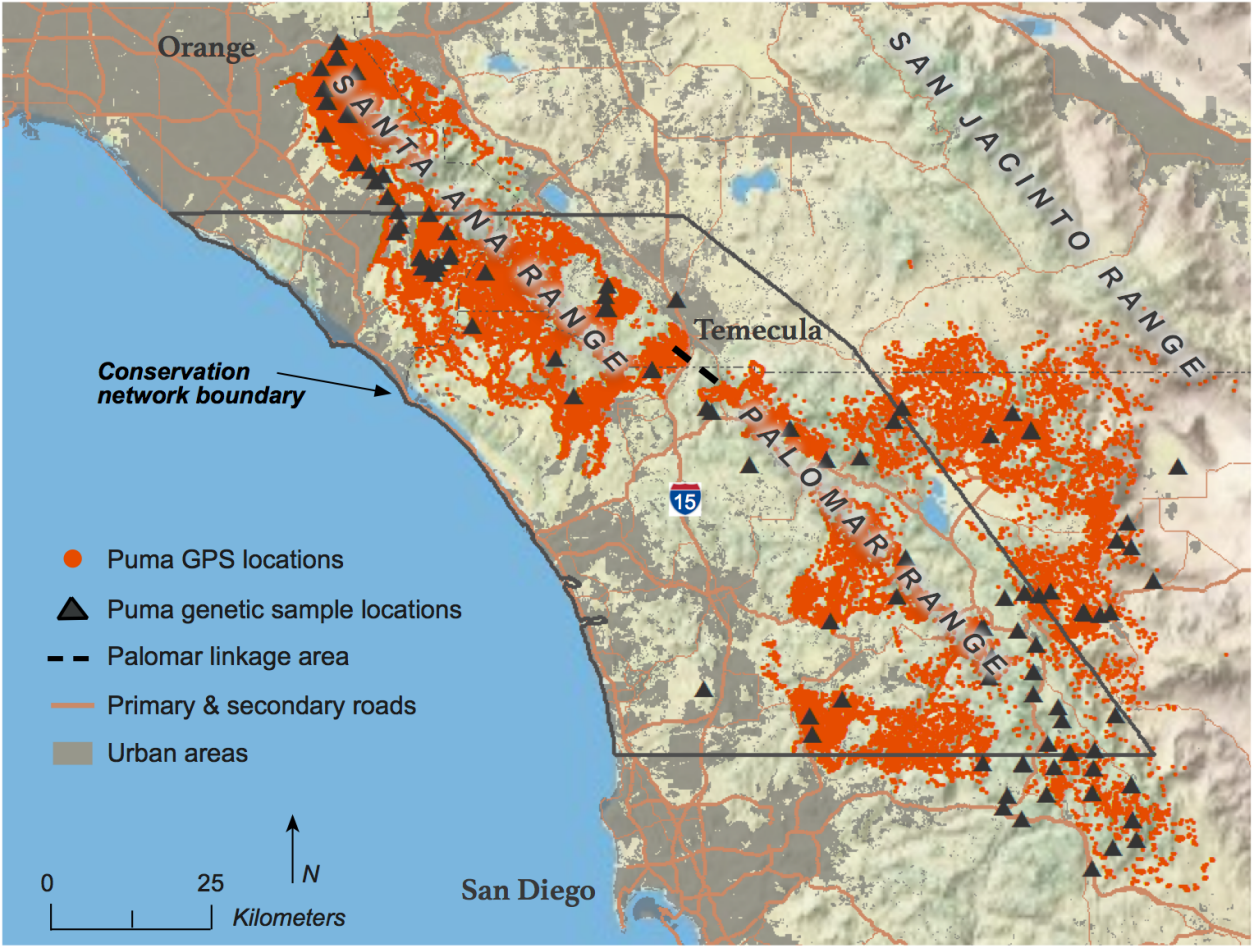
Southern California study area and conservation network sub-area with puma GPS telemetry and genetic sample locations used in the analysis.

The study area was located across much of the Peninsular Mountain Range in southern California spanning from southern Los Angeles and San Bernardino Counties in the north to the U.S.–Mexico border in the south (Fig 1). Interstate 10 and the Salton Sea approximately defined the eastern boundary and the Pacific Ocean was the western boundary. The region is defined by a Mediterranean climate with hot dry summers and mild, wetter winters. Elevations range from sea level to 3200 m. Outside of urban areas vegetation is primarily composed of chaparral, coastal scrub, grasslands, and oak woodlands.

Between 2005–2015, pumas throughout the study area were captured and fit with GPS collars under California Fish and Wildlife Scientific Collecting Permit number 9875 and University of California Davis Institutional Care Use Committee authorization number 17233. Please see Vickers et al. [27] for capture details and protocols. Collar acquisition interval varied from five minutes to six hours. To avoid the use of data that may have large spatial errors, we removed two-dimensional fixes with a Position Dilution of Precision > 5 [28].

### GIS data and predictor variables

We used predictor variables that have been shown to influence puma habitat use and movement. These included: elevation, percent slope, terrain ruggedness (calculated as total curvature in DEM Surface Tools; [29]), roads, urban areas, and land cover types aggregated for the study area [13, 30–32]. Variable details are provided in Table 1. All variables were represented with a 30 m spatial resolution.

**Table 1:** Predictor variables used in the puma resource selection, movement selection, and landscape genetic analyses.

Variable	Source/Derivation	Year
***Topographic features***		
Elevation	USGS National Elevation Dataset	2009
Percent Slope	Derived from National Elevation Dataset	-
Terrain Ruggedness	Total curvature derived from National Elevation Dataset with DEM Surface Tools [29]	-
***Land cover type***		
Agriculture	Aggregated agricultural classes from CalVeg	2014
Chaparral	Aggregated chaparral classes from CalVeg	2014
Coastal Scrub	Aggregated scrub-type classes from CalVeg	2014
Coastal Oak Woodland	Aggregated woodland classes from CalVeg	2014
Grassland	Aggregated grassland classes from CalVeg	2014
Barren/Open Water	Aggregated barren and water classes from CalVeg	2014
Desert	Aggregated desert classes from CalVeg	2014
Riparian	Aggregated riparian classes from CalVeg	2014
***Human development***		
Urban	National Land Cover Data	2011
Roads; Classified as Primary, Secondary, and Tertiary	U.S. Census Bureau TIGER	2014
Roads; Classified as Primary, and Secondary	U.S. Census Bureau TIGER	2014
Roads; Classified as Primary	U.S. Census Bureau TIGER	2014

### Level II resource selection: Home range selection function

Because our study area is well within the geographic range of pumas [33], we assumed that probability of use was 100% for Level I selection. Therefore, we focused only on Levels II and III for our resource selection analyses.

All data analysis was conducted in the R software environment [34]. Home Range Selection Functions (HRSFs) were conducted in the same ‘used’ and ‘available’ framework typical of RSFs [35], where points distributed within empirical home ranges are the ‘used’ data and points distributed within a buffer around the empirical home ranges are the ‘available’ data. To estimate puma home ranges, we used pumas with at least five months of data. Based on prior analyses by Grigione et al. [36] and our observations of pumas in the study area, there is little variation in home range size among seasons, therefore we are confident five months is a sufficient amount of time to capture the full home range utilization of individuals. Data were visually examined to determine if individuals moved from their original home ranges to new home range areas during the study period. If home range shifts were identified, we excluded these individuals from the analysis. This resulted in a total of 31 pumas for the HRSFs (Males = 12, Females = 19) with collar duration ranging from five months to 32 months (mean = 10.87 months).

We followed the recommendations of Borger et al. [37], Bauder et al. [38], and Fieberg [39] to estimate puma home ranges and standardized our sampling regime among individuals by using only points from the longest collar fix interval (six hours) to reduce home range estimation bias. This subsetting resulted in a total of 24,911 locations with a per-individual mean of 811 points (range = 284–1535).

We used the *ks* package in R [40] to estimate kernel density home ranges using the reference bandwidth and an unconstrained bandwidth matrix. The unconstrained bandwidth matrix has been shown to produce a single volume contour, to encompass most of the input points, and to be less sensitive to fix rate and sampling duration than other home range estimators [38]. We identified our final home range boundaries as 90% of the entire utilization distribution for each individual.

For our used data, we randomly distributed points throughout the each puma home range. The number of points selected equaled the number of points used to estimate the home- range kernels. For our available data, we first calculated the maximum distance between points for each home range and identified the 95^th^ quantile of this distribution. We then buffered all used home range points by this distance (43 km), which constituted our available area. Unique available points were randomly distributed throughout this available area in a 1:1 ratio with the used points.

We developed multi-scale HRSFs using a two-stage, pseudo-optimized approach [2]. We calculated our used and available data within 10 ecological neighborhoods of varying sizes (50 m, 100 m, 200 m, 500 m, 1000 m, 2000 m, 4000 m, 6000 m, 8000 m, 10000 m). These neighborhoods were weighted by a Gaussian kernel using the ‘kernel2dsmooth’ function from the *smoothie* package in R [41]. In the first stage, we ran univariate logistic regression models to identify the characteristic scale of selection for each landscape variable as indicated by the lowest Akaike Information Criterion value corrected for small sample size (AICc; [42]). In the second stage, we combined the optimal scales for each landscape variable into a multiple logistic regression model (omitting the lesser performing variable of any pair that had a correlation greater than 0.6). Because we thought all the variables would have some influence on puma habitat use, we then used the ‘dredge’ function in package *MuMIn* [43] to run all variable subsets and identify the best model.

### Level III resource selection: Point selection function

We used the same individuals and GPS point locations in the Point Selection Function (PSF) as we used to estimate puma home ranges. We estimated the used data as the proportion (for categorical data) or mean (for continuous data) of each predictor variable within a 30 m uniform buffer around each GPS location. We estimated the available data within a larger ecological neighborhood around each used point weighted by a Pareto kernel. The Pareto kernel was derived from our empirical movement data (see Zeller et al. [21] for details). The used and available data were analyzed in a paired, or conditional, logistic regression framework [44]. Therefore, each used point was paired with a biologically relevant available area. This approach, also referred to as a context-dependent PSF, estimates habitat selection at each location across the study area based on its location and surrounding environment and allows us to model Level III habitat use [5, 21].

We examined multiple scales by varying the size of the Pareto kernel for estimating available. Nineteen different sizes of the Pareto kernel were determined by using the 5-min data to create empirical movement distributions. Specifically, we used the 5-min GPS data to calculate movement distances over a 5-min time period and fit a Pareto distribution to this empirical distribution using the *gPdtest* package [45]. We then subsetted the data at progressively longer time periods up to 6-hr and re-fit the Pareto to each of these empirical distributions of movement distances. This resulted in 19 Pareto kernels with the following distances representing 95% of the distribution: 241 m, 408 m, 681 m, 915 m, 1123 m, 1317 m, 1602 m, 1850 m, 2049 m, 2298 m, 2312 m, 2797 m, 3044 m, 3104 m, 3479 m, 3819 m, 3994 m, 4099 m, and 4461 m. The available data were calculated as the proportion (for categorical data) or mean (for continuous data) of each predictor variable around each used point weighted by each Pareto kernel. Movement time periods and estimated Pareto distribution parameters are provided in S1 Table.

We developed our multi-scale models using the same two-stage approach as described for the HRSFs. We ran the paired logistic regression models with the ‘coxph’ function in the *Survival* package [46]. We had attempted to run these models in a mixed-effects framework to account for individual variability, but had model convergence issues. To compensate for the lack of an individual level mixed-effect, we used robust standard errors, which are calculated by combining data into clusters such that the clusters are not autocorrelated [47–49]. Robust errors are often used to control for the individual level effects in paired regression models [47, 50]. We ranked the models using AICc and arrived at our final model by averaging any models that had a ΔAICc ≤ 2 from the best model. We used the robust standard errors when calculating confidence intervals for the model-averaged coefficients.

### Estimating resistance from movement data

We performed Path Selection Functions (PathSFs) to estimate the relative probability of movement for pumas across the study area. Our previous work has shown that PathSFs are sensitive to fix interval and that, for pumas, biases are introduced with fix intervals of 1-hour and greater [13]. Therefore, for this analysis we used data from individuals with either a 5-min or 15-min fix interval for at least a 2-week duration. We created paths for each individual by connecting consecutive points for each 24-hour time period. This resulted in a total of 39 pumas (Males = 20, Females = 19) and 1,076 daily paths for the PathSF analysis (mean per individual = 30 days, median = 22 days, range = 14–106).

We used the same resource selection approach as for the PSFs above, but instead of points as our unit of inference, we used daily paths. The previously described scales and two-stage approach were used to develop multi-scale PathSFs.

### Estimating resistance from genetic data

Between 2001–2016, blood or tissue samples were collected from 146 captured or deceased pumas across the greater study area. Nuclear DNA was extracted and characterized at 44 microsatellite loci, which met the assumptions of Hardy-Weinberg proportions and linkage equilibria as described in Gustafson et al. [51]. Of the 146 individuals, 139 were located in the study area and used for this analysis (Fig 1).

**Fig 2:**
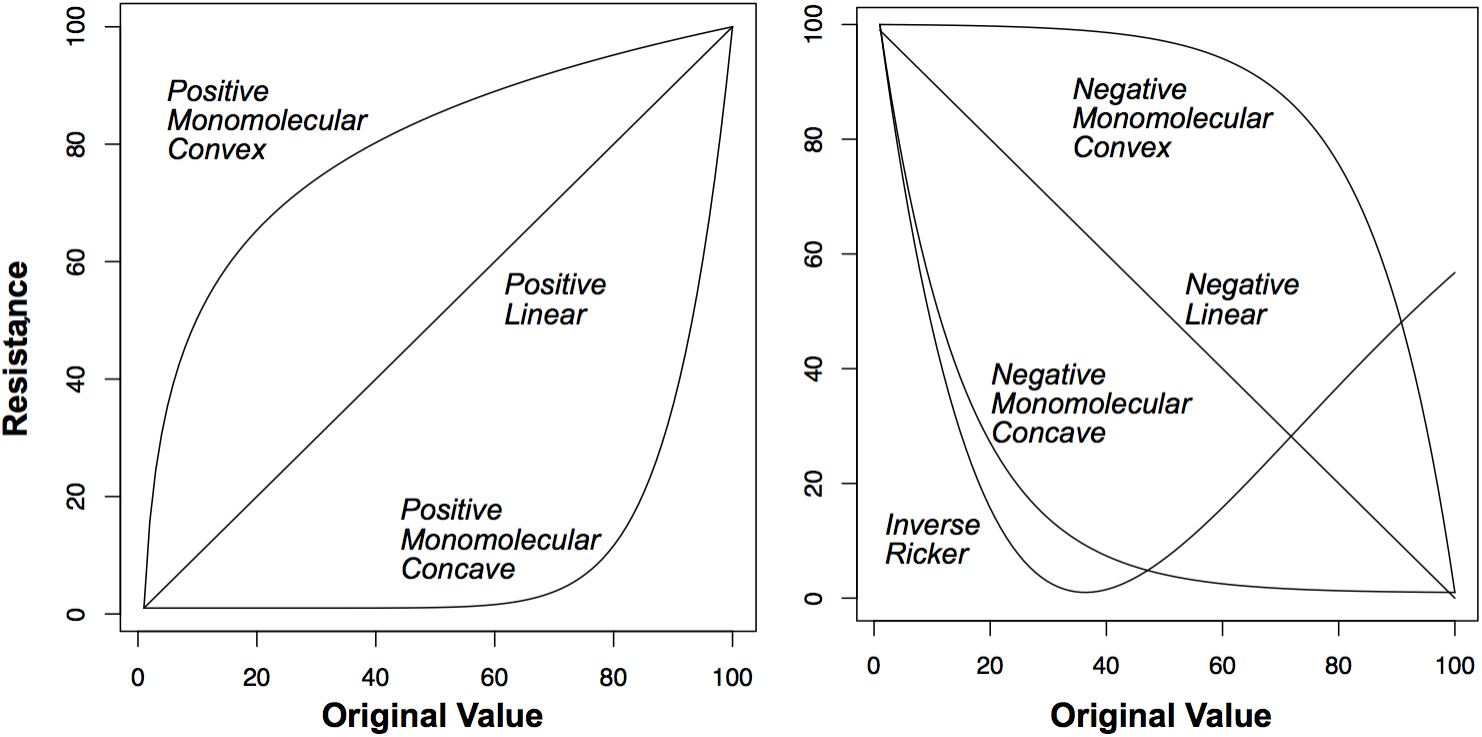
Functions used to transform the environmental variables to resistance, with a range of 1–100, for use in the landscape genetic analysis.

Landscape genetic approaches aim to correlate observed genetic distances among individuals or populations with geographic distances. These geographic distances are calculated as the least-cost distance or resistance distance among individuals across resistance surfaces defined *a priori*. We explored a number of different resistance hypotheses for each of our predictor variables. Specifically, we represented each variable with four ecological neighborhoods (100 m, 500 m, 2000 m, and 6000 m) weighted by a Gaussian kernel. Computational capacity limited us from testing a wider range of scales; therefore, we selected an array of scales we thought was biologically appropriate for this analysis. We then applied each of seven functions to transform the scaled variable into a resistance value of 1–100 (Fig 2). Positive or negative transformation functions were used to represent increasing or decreasing resistance with increasing values of that variable, respectively. We also used the inverse Ricker transformation to account for variables that might have a low resistance at moderate values. Therefore, for each variable we tested a suite of 28 *a priori* resistance surfaces.

With the *adegenet* package [52], we calculated pairwise genetic distances among all 139 individuals using Nei’s genetic distance [53]. We calculated pairwise geographic distance by calculating the least cost path distance between all sample locations across each *a priori* resistance surface with the *gdistance* package [54]. We then compared all the *a priori* resistance surfaces for a variable by running univariate linear mixed effects models that accounted for the pairwise structure of the distance matrices following the maximum likelihood population-effects (MLPE) method [55, 56]. This method has recently been shown to outperform other correlational methods in landscape genetics such as Mantel tests or multiple regression with distance matrices [57].

We used AICc to identify the most appropriate resistance surface for each variable. We assessed correlations among variables and removed variables from correlated pairs with higher AICc values. We then ran multiple regression models with all uncorrelated variables and fit all possible subsets of the variables. We ranked the multiple regression models using AICc and identified our top model.

### Multi-level resource selection function and resistance surfaces

We predicted the relative probability of use across our study area from the HRSF by using the following formula $\hat{w}\;(x)=exp(\beta_{1}x_{1}+\beta_{2}x_{2}+\beta_{3}x_{3}+\ldots +\beta_{p}x_{p})$ as recommended by Johnson et al. [35]. We predicted the relative probability of use from the PSFs and PathSFs in the paired framework described by Zeller et al. [13]. This approach requires ‘used’ and ‘available’ to be calculated for every pixel in the study area. We first calculated the proportion or mean of each variable within a 30 m fixed-width buffer at each pixel in our study area. We then calculated the proportion or mean of each variable at each pixel weighted by the Pareto kernel at the appropriate scale for that variable. This is akin to the ‘used’ and ‘available’ in the paired regression models and allows for a unique relative probability of movement to be identified for each pixel in the study area.

We converted the relative probabilities of the PathSF surface to resistance by subtracting the relative probabilities of movement from one and multiplying by 100. For the landscape genetics analysis, we derived our resistance surface by summing the resistance surfaces for the variables in the final model and rescaling from 1–100.

We multiplied the predictive surfaces from the Level II HRSF and the Level III PSF and rescaled the surface from 0–1 to obtain a single multi-scale ML-RSF. We quantile re-scaled the landscape genetics and PathSF resistance surfaces, multiplied them, and rescaled the surface from 1–100 to obtain a single multi-scale ML-RS.

### Resource use patches and corridors

The greater Northern San Diego County area (including parts of southern Orange and Riverside Counties) was the focal landscape for our conservation plan. All further analyses were conducted on this subset of our original study area (Fig 1; Conservation Network Boundary).

To identify resource use patches, we first smoothed the ML-RSF surface using a Gaussian kernel with a bandwidth of 500 m. We then identified patches on this surface that had at least a 60% probability of resource use and were at least as large as the minimum observed female home range size.

To model corridors, we first probabilistically distributed 2000 source points on the ML-RSF surface within the resource-use patches. We then used UNICOR software [58] to identify resistant kernel corridors [9] across our ML-RS surface from the source points. Resistant kernels allow the identification of a maximum dispersal distance, which we set to 100 km for puma [27]. We identified landscape corridors by taking the top 75% of the resistant kernel surface.

We calculated the percent overlap between our conservation network (resource use patches and corridors) and the current and proposed protected area network to highlight gaps in protection for pumas and identify areas for future conservation attention. Current protected areas were identified from the California Protected Areas Database [59]. We considered Department of Defense, Native American Reservation, and large Water and Irrigation District lands as partially protected and included these as a separate category in our calculations. Proposed protected areas were lands designated as Pre-Approved Mitigation Areas by Orange, Riverside, and San Diego Counties.

## Results

### Level II resource selection: Home range selection function

Home range sizes varied from 41–497 km^2^ (Mean = 231 km^2^; Female mean = 188 km^2^; Male mean = 316 km^2^). Characteristic scales differed across predictor variables (Table 2), but pumas generally selected for coarser scales when establishing home ranges. Due to convergence errors, we were unable to fit the models for primary roads. We identified a single best model for puma home range selection (Table 3). This model indicates pumas select for home ranges with more rugged terrain, naturally barren areas, chaparral, coastal scrub, and grassland than the surrounding landscape and fewer areas of agriculture, desert, woodland, and urban.

**Table 2:** Characteristic scales of selection for each predictor variable from the Level II and Level III selection functions, the Path selection functions, and landscape genetic analysis. Plus or minus indicates preference or avoidance of that variable for resource use or movement. The selected resistance transformation for the landscape genetic analysis are indicated by IR = inverse Ricker, NL = negative linear, NMCc = negative monomolecular concave, NMCv = negative monomolecular convex, PL = positive linear, PMCc = positive monomolecular concave, PMCv = positive monomolecular convex. Blank cells indicate model convergence failures.

Variable	Level II selection function		Level III selection function		Landscape genetics analysis		Path selection function	
	*Scale (m)*	*Sign*	*Scale (m)*	*Sign*	*Scale (m)*	*Trans-formation/ Sign*	*Scale (m)*	*Sign*
***Topographic features***								
Elevation	2000	+	241	-	6000	IR	241	+
Percent slope	8000	+	241	-	100	NMCc +	2797	-
Terrain ruggedness	10000	+	4461	+	500	IR	681	+
***Land cover type***								
Agriculture	6000	-	4461	-	6000	PL -	3819	-
Chaparral	4000	+	241	-	6000	NMCc +	3104	-
Coastal Scrub	500	+	681	-	500	NMCv +	241	-
Coastal oak woodland	10000	+	4461	+	2000	NMCv +	241	+
Grassland	10000	+	4461	-	500	PMCv -	2797	-
Barren/Open water	4000	+	3994	-	100	NL +	3104	-
Desert	8000	-			6000	PMCc -		
Riparian	10000	+	3497	+	500	NMCv +	1317	+
***Human development***								
Urban	2000	-	4461	-	500	PMCv -	241	-
Roads; Classified as Primary, Secondary, and Tertiary	10000	-	4461	-	500	PMCv -	3819	-
Roads; Classified as Primary, and Secondary	6000	-	4461	-	2000	PL -	4461	-
Roads; Primary only					500	PMCv -		

**Table 3:** Standardized beta estimates, robust standard errors, and 95% robust confidence intervals for the multivariate Level II Home Range Selection Function model variables.

Variable	Beta estimate	Standard Error	95% Confidence Interval
Terrain ruggedness	0.95	0.01	0.94–0.96
Agriculture	-0.04	0.01	-0.05 –-0.03
Barren	0.19	0.01	0.18–0.20
Chaparral	0.17	0.02	0.15–0.18
Coastal oak woodland	-0.05	0.01	-0.06 –-0.05
Coastal scrub	0.15	0.02	0.14–0.16
Desert	-1.26	0.04	-1.28 –-1.23
Grassland	0.11	0.02	0.1–0.12
Urban	-0.68	0.03	-0.70 –-0.66

### Level III resource selection: Point selection function

Univariate model results indicate pumas had a mostly bi-modal response to landscape features at the third order of habitat selection (Table 2). Pumas responded to elevation, percent slope, chaparral, and coastal scrub at fine scales and responded to the other variables at coarse scales. Due to convergence errors, we were unable to fit the models for desert and primary roads.

After removing correlated variables, the global model was identified as the top model. Pumas preferred slightly more rugged terrain, riparian areas and woodland while avoiding high elevation, high slopes, agriculture, barren, chaparral, coastal scrub, grassland, urban, and primary, secondary, and tertiary roads (Table 4).

**Table 4:** Standardized beta estimates, robust standard errors, and 95% robust confidence intervals for the multivariate Level III Point Selection Function model variables.

Variable	Beta estimate	Standard Error	95% Confidence Interval
Elevation	-21.61	0.60	-21.99 –-21.23
Percent Slope	-1.1	0.03	-1.12 –-1.08
Terrain Ruggedness	0.09	0.01	0.08–0.09
Agriculture	-0.25	0.02	-0.23 –-0.26
Barren	-0.06	0.02	-0.05 –-0.07
Chaparral	-0.17	0.06	-0.21 –-0.13
Coastal Scrub	-0.29	0.03	-0.03 –-0.27
Grassland	-0.38	0.02	-0.40 –-0.37
Riparian	0.38	0.04	0.35–0.40
Woodland	0.23	0.02	0.22–0.24
Urban	-2.18	0.16	-2.28 –-2.08
Roads: Primary, Secondary, Tertiary	-0.06	0.02	-0.07 –-0.05

### Estimating resistance from movement data

Pumas selected for more landscape variables at finer scales during movement than during resource selection (Table 2). After removing correlated variables, four top models were identified and beta coefficients were averaged. Pumas also showed more tolerance of landscape variables during movement than during resource-use events. Pumas avoided steep slopes, agricultural areas, urban areas, and roads during movement, but showed a preference for all other landscape variables in the final model, especially riparian and woodland areas (Table 5).

**Table 5:** Standardized beta estimates, robust standard errors, and 95% robust confidence intervals weights for the multivariate Path Selection Function model variables.

Variable	Beta estimate	Standard Error	95% Confidence Interval
Elevation	9.22	1.00	8.51–9.94
Percent Slope	-1.35	0.21	-1.50 –-1.20
Agriculture	-0.02	0.09	-0.08–0.05
Chaparral	1.44	0.30	1.37–1.51
Grassland	-0.02	0.28	-0.22–0.18
Barren/Open Water	-0.02	0.07	-0.07–0.04
Riparian	5.92	1.90	4.56–7.27
Woodland	2.87	0.36	2.61–3.13
Urban	-7.53	2.03	-8.98 –-6.08
Roads: Primary, Secondary, Tertiary	-0.78	0.24	-0.95 –-0.62

### Estimating resistance from genetic data

The linear mixed effect models resulted in identifying the characteristic scale and transformation for each variable in relation to the genetic distances among individuals (Table 2). Variables whose resistance increased with increasing values were agriculture, grassland, urban, and roads. Variables whose resistance decreased with increasing values were chaparral, percent slope, riparian, coastal scrub, and coastal oak woodland. Resistance for elevation and ruggedness were represented by an Inverse Ricker transformation, which decreases until middle values are reached, and then increases for the remaining values, indicating dispersal is facilitated at mid-elevation and mid-ruggedness values.

After accounting for correlations, the following variables were included in the multiple regression model: elevation, percent slope, agriculture, chaparral, coastal scrub, coastal oak woodland, grassland, riparian, urban, and primary roads. The global model was selected as the best performing model. To determine whether the variables explained the genetic distance among individuals more than Euclidean distance alone, we also ran a regression model with a simple Euclidean distance matrix among sample locations. This resulted in a ΔAICc of 278, which was much higher than any other model, indicating the environmental variables explained the genetic distance among individuals better than Euclidean distance alone. To determine whether long-term genetic processes were driven largely by fine scale movement data, we also calculated geographic distances among sample locations across our PathSF resistance surface and obtained low model support (ΔAICc = 247), indicating different drivers for long-term genetic connectivity.

### Multi-level resource selection function and resistance surfaces, resource use patches, and corridors

**Fig 3:**
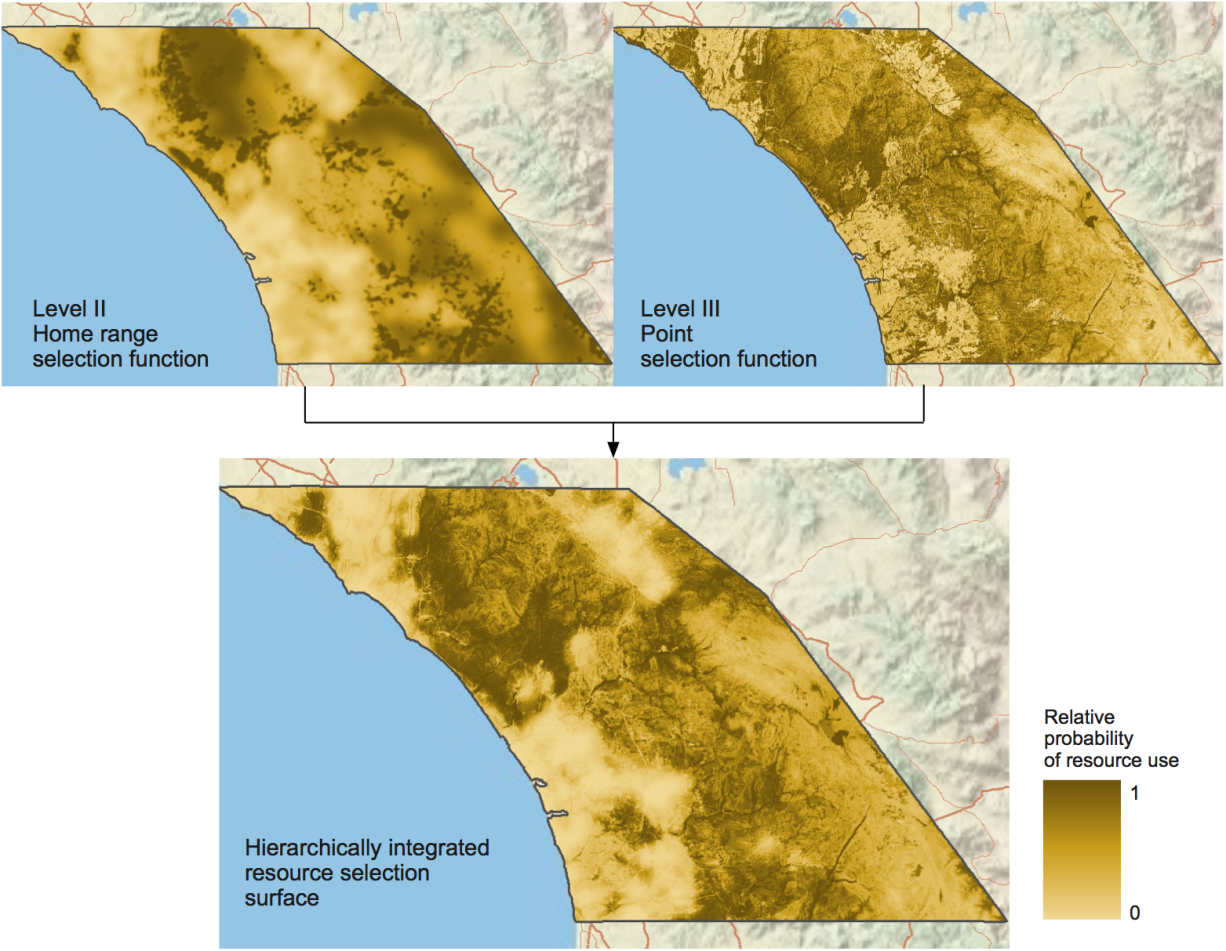
Predicted relative probability of use surfaces from the multi-scale Level II Home Range Selection Function, the multi-scale Level III Point Selection Function and the combined Multi-Level Resource Selection Function.

Fig 3 presents the predicted relative probability of use surfaces for the HRSF, the PSF, and the combined ML-RSF. Home range centers tended to be located further from urban areas and in more mountainous terrain. The Level III selection surface also tended to select for non-urban areas, but this surface was more heterogeneous than the Level II surface, demonstrating finer-scale selection. The ML-RSF surface identified areas of Level III habitat selection conditional upon the higher Level II order of selection.

**Fig 4:**
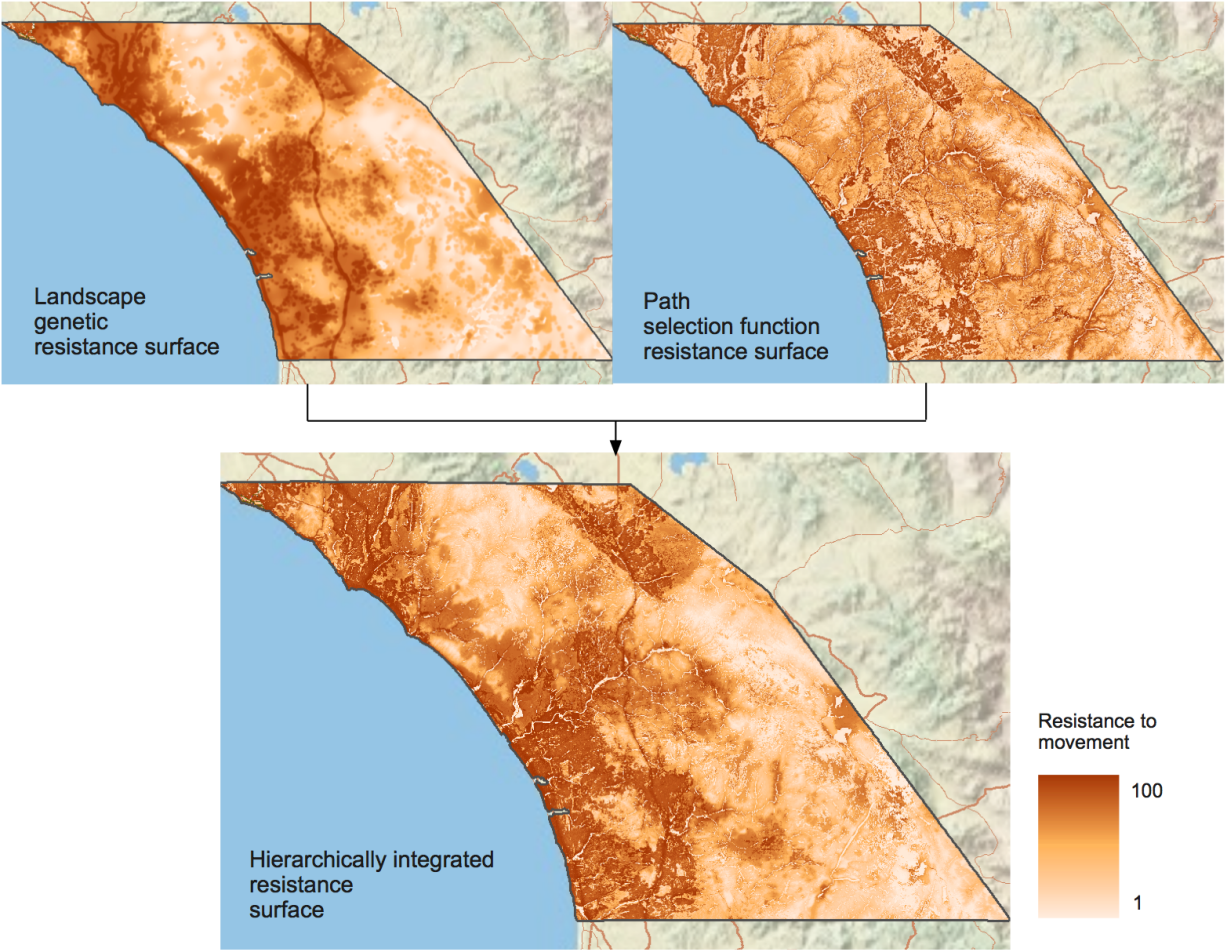
Resistance surfaces derived from the landscape genetics analysis, the PathSF, and the combined Multi-Level Resistance Surface.

Fig 4 displays the resistance surfaces derived from the landscape genetics analysis, the PathSF, and the ML-RS. Similar to the probability of use surfaces at the higher order, the landscape genetic analysis resulted in developed areas having a high resistance for pumas and undeveloped, mountainous areas having a lower resistance. Though the coarse-scale patterns were similar for the resistance surface derived from the PathSF, this surface was more heterogeneous and reflected finer-scaled patterns of resistance to movement. The Spearman correlation coefficient between the landscape genetics and the PathSF resistance surfaces was 0.31. The ML-RS can be interpreted as resistance to movement at finer scales weighted by coarser-scale spatial and temporal processes.

**Fig 5:**
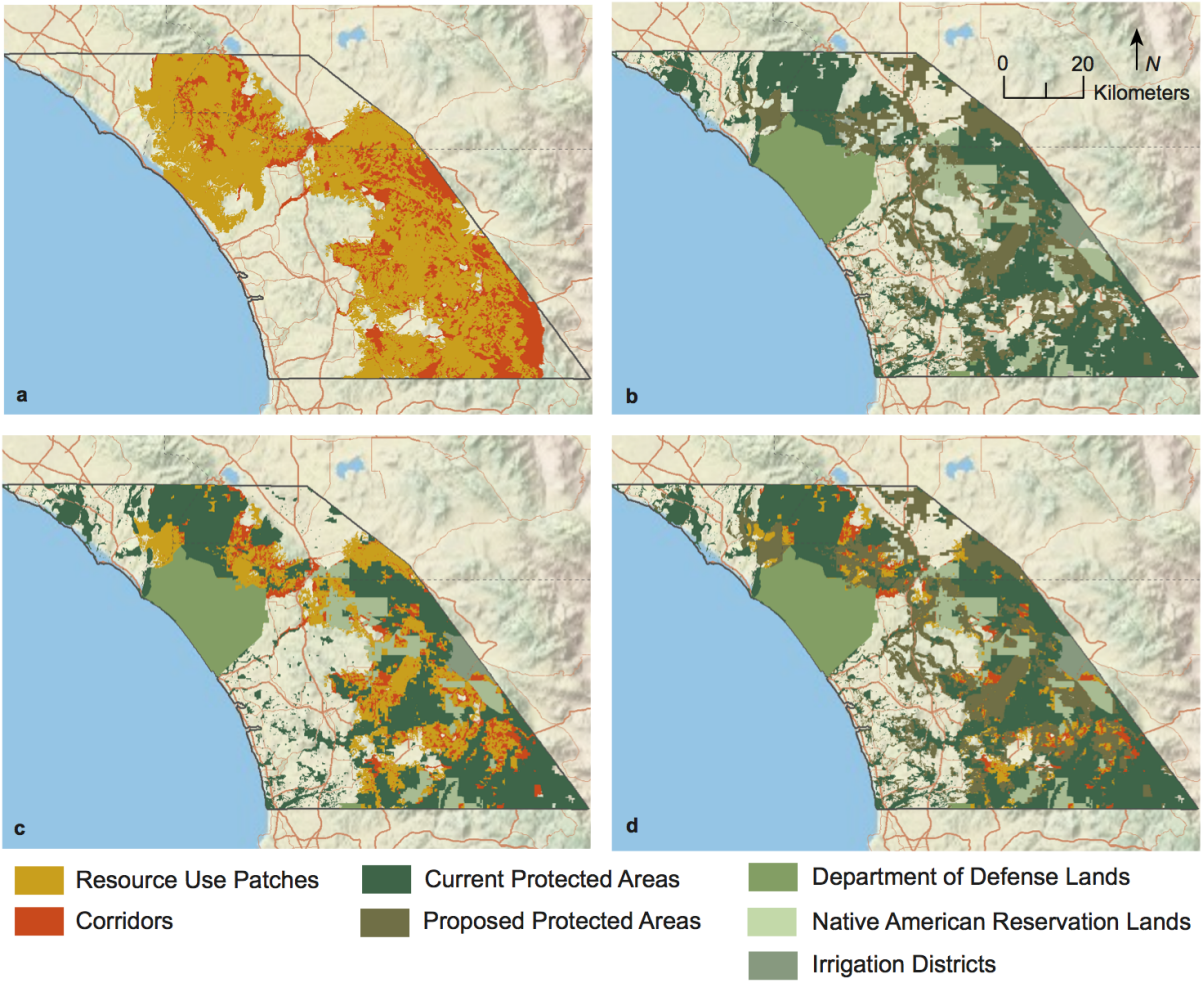
Puma resource use patches, landscape corridors, and the current and proposed protected area network.

Resource-use patches in the study area measured 2,577 km^2^. Resistant kernel corridors connecting these resource-use patches measured 777 km^2^ (Fig 5A). The current protected area network encompassed 35% of the puma resource-use patches in the study area and 47% of the landscape corridors outside of the resource use patches (Fig 5C). Adding the partially protected Department of Defense, Native American Reservation, and Water and Irrigation District lands increased current protection of resource-use patches to 61% and landscape corridors to 60% (Fig 5C). If 75% of the lands that are currently pre-approved for mitigation protection by Orange, Riverside, and San Diego Counties become protected (the 75% level is the target amount), then 88% of the puma resource-use patches will be fully or partially protected and 82% of the landscape corridors will be fully or partially protected (Fig 5D).

## Discussion

Multi-level, multi-scale resource selection functions and resistance surfaces capture a wide spectrum of selection, movement and survival process. Multi-level, multi-scale resource selection models have been shown to outperform single-scale, single-level models, result in stronger inference and predictive capabilities [6, 7, 17, 21], and are the most appropriate for use in conservation planning [60]. However, to our knowledge, the multi-level, multi-scale framework had not been applied to resistance surfaces. Herein, we introduce a multi-level, multi-scale approach for developing resistance surfaces. Our ML-RS quantifies resistance in terms of all three previously sought-after processes: (1) willingness to cross, (2) physiological costs of crossing, and (3) direct survival impacts while crossing. When establishing landscape-level wildlife corridors, the ultimate objectives are to provide safe passage for migrants to access, establish residence, and successfully reproduce in new suitable habitats. Resistance surfaces that reflect real-time movement and decision-making of individuals, as well as the chances of surviving and reproducing, are conceptually more powerful than resistance surfaces that reflect only one of these three processes.

Our results showed similar coarse-scale patterns between the resistance surfaces derived from the genetic and movement data; however, details were present in the genetic surface that were not present in the movement surface, and vice versa. For pumas in our study, the resistance surface derived from the movement models consistently showed primary roads and urban areas as less-resistant features than the resistance surface derived from genetic data. These results reflect the ability and occasional willingness of pumas to travel through highly developed areas. Conversely, the movement data highlighted the importance of riparian areas and canyons for puma movement, assigning consistently lower resistance values to these features than the resistance surface derived from genetic data. Combining these surfaces resulted in constraining fine-scaled movement (potential resistance) within areas more amenable to coarse-scale, generational success in dispersal and breeding (realized resistance).

We can further illustrate the advantages of a ML-RS through our collective knowledge of the Santa Ana Mountains puma population. This population inhabits what has been described as a large habitat fragment, isolated on all sides by roads, human development, and the Pacific Ocean [25]. The Palomar Linkage (Fig 1.; [61]) just south of the city of Temecula has been identified as the only potentially viable linkage between the Santa Ana Mountain puma population and those to the east. The Palomar Linkage connects natural areas on both sides of I-15, yet I-15 itself is a significant obstacle to puma movement. Genetic population assignment tests have shown that only seven males successfully crossed I-15 over the last 15 years; three immigrated into the Santa Ana Mountains from the Eastern Peninsular Range and four traveled from the Santa Ana Mountains to the Eastern Peninsular Range [51]. Of these seven, five are known to be deceased and the status of the remaining two is unknown. Offspring were only detected from one of the seven immigrants, M86, who migrated into the Santa Ana Mountains. M86 successfully bred and passed genetic material to 11 offspring before being hit by a car and killed. Of the 11 offspring, only two are confirmed to have survived to breeding age, yet the success of this one male was shown to significantly improve the genetic diversity of the Santa Ana Mountains population [51]. The seven males that crossed I-15 offer evidence of the occasional ability and willingness of pumas to travel through urbanized areas and across primary roads. The behavior of these individuals is therefore better represented with the movement resistance surface. In contrast, the physiological and survival costs of these movements, exemplified by the low survival and breeding success of these seven pumas, are better reflected with the genetic resistance surface. The combination of these surfaces into a single ML-RS integrates all three of these processes and is a more appropriate representation of resistance for identifying landscape-level connectivity.

Consistent with other studies that conducted resource selection functions at multiple hierarchical levels, we found differences among levels in terms of variable importance and habitat preference for pumas [2, 4, 5, 7]. Of the variables present in both the Level II and Level III models, habitat use relationships differed for chaparral, coastal scrub, coastal oak woodland, and grassland. Chaparral, scrub, and grassland types were preferred for home range establishment, but were avoided while using resources within the home range. Our results for woodland were unexpected. Pumas are traditionally associated with woodland, but our Level II model showed an avoidance of woodland. We suspect this is the result of a relatively low occurrence of woodland in the Santa Ana and Palomar Mountain ranges compared with a higher amount of woodland in the greater extent used for the HRSF analysis. Therefore, this finding is likely an anomaly of study area extent and does not indicate a true avoidance [62]. As expected, woodland was preferred in our Level III model. Variables that were included in the final Level III model that were not present in the Level II model include primary, secondary, and tertiary roads, elevation, slope, and riparian areas. With the exception of woodland at the second order of selection, our results generally agree with other puma resource-use studies. We found topographic variables to be important at both hierarchical levels with a strong preference for rugged areas and an avoidance of areas with steep slopes [25, 30, 32]. We also found a strong avoidance of human development in the form of agricultural areas, urban areas, and roads [21, 30–32]. Home range sizes were also consistent with other studies of pumas in Mediterranean climates [63].

Similar to our findings across hierarchical levels of selection, we found some differences between the resistance models derived from the movement and genetic data. The genetic model included coastal scrub whereas the movement model did not and instead included the barren cover type. The genetic model only included primary roads whereas the movement model included the roads layer with all three road types: primary, secondary, and tertiary. For some variables, the models differed in their relationship with resistance. For example, the genetic model reflected decreasing resistance with increasing values of slope, and the movement model reflecting increasing resistance with increasing values of slope. For elevation, comparison is more difficult. The genetic resistance model selected the Inverse Ricker transformation, indicating lower resistance at moderate elevations and higher resistance for very low and high elevation areas. Given the paired nature of our PathSF model, we were unable to explore quadratic relationships and the resistance surface from the movement data reflected decreasing resistance with increasing elevation. Cushman and Lewis [10] compared black bear genetic models with early season movement models and found both to have the same variables with the same relationship to resistance, but observed different variables and different relationships when comparing genetic models with late season movement models. Coulon et al. [15,16] found roe deer exhibited different relationships to woodland areas with genetic and movement data. These differences are to be expected because, as argued above, resistance estimated from genetic data and resistance estimated from movement data represent different processes. This is further supported by our landscape genetic model performance results. We found the final resistance surface derived from the landscape genetic analysis to greatly outperform that derived from the PathSF analysis in predicting genetic distance, indicating potentially different drivers for long-term genetic connectivity.

Another possible advantage of combining the resistance surfaces from movement and genetic data lies in the difficulty of obtaining dispersal data. It is often argued that dispersal data are more appropriate than data from within home-range movements for estimating resistance; however, dispersal data are much more difficult to obtain and often suffer from very small sample sizes. By using genetic data as a proxy for dispersal, and combining this resistance surface with one derived from within home-range movement, these surfaces may more closely approximate those obtained with empirical dispersal data, though more research is needed to support this hypothesis. We acknowledge the ML-RS may not be the most appropriate resistance surface to use for every application and recommend its use for landscape-level corridors only. For other applications, like pinpointing discrete road crossing locations or applications that reflect fine-scaled travel and real-time movement decisions, we argue that it is more appropriate to use a resistance surface estimated directly from movement data [11].

Our conservation network for pumas in the study area incorporated multiple scales of selection at two different hierarchical levels for both habitat use and resistance. In this heavily developed landscape, we found resource use and connectivity to be mostly confined to natural areas with only corridors extending into more developed areas. At present, only 35% of resource-use patches and 47% of corridors identified in this study are fully protected. Some additional lands offer partial protection, bringing the fully and partially protected lands to about 60% of the resource-use patches and corridors. Given the low adult survival rate (56%; [27]), low levels of heterozygosity and high incidence of inbreeding for pumas in this region [26], increasing protection of individuals and habitat is essential to their long-term survival [64]. The proposed protected areas identified by the three counties in the study area would greatly increase the amount of protection. However, many of these proposed protected areas are currently on private land and depend upon them being used as mitigation for development elsewhere. Road mitigation efforts are also needed given the low level of successful dispersal and breeding among pumas in the study area and the finding in Vickers et al. [27] that the most frequent cause of death for GPS-collared pumas was by vehicle collisions.

Our analysis was specific to pumas in southern California, yet the general approach may be applied to wildlife species worldwide. Many previous conservation networks have used a single organizational level, thereby omitting important behavioral and biological processes at other levels, and may result in incorrect inference and only partially effective conservation plans [2, 5, 7, 65]. By incorporating selection and movement at multiple hierarchical levels, correct inference may be made at each level. Furthermore, integrating results across levels will result in much stronger predictive surfaces for conservation. Modeling multi-scale relationships within each hierarchical level further strengthens these surfaces. Our thresholds for identifying the resource-use patches and landscape corridors were somewhat arbitrary, but are based on previously recommended thresholds [66] and provide a reasonable result for pumas in our study area. Echoing DeCesare et al. [4], additional research is needed to determine more biologically-based thresholds for creating binary conservation surfaces from continuously distributed ML-RSFs and connectivity surfaces derived from ML-RSs. Despite the strong conceptual and inferential advantages of multi-level, multi-scale approaches, a recent review of 173 multi-scale habitat selection studies found that only 8 (5%) used a combined multi-level, multi-scale approach, indicating this approach for research and conservation planning has been underutilized [2]. We offer the addition of multi-level, multi-scale resistance surfaces to this body of literature and recommend that multi-level, multi-scale approaches be used for identifying areas of resource use and landscape connectivity and for developing species conservation networks.

## Supporting information

S1 Table Time-intervals and associated radii of Pareto kernels used to define available habitat for point and path selection functions. We fit a Pareto distribution to the empirical distribution of displacement distances at each time-period and defined the maximum radii of the Pareto distribution by either using the 95^th^ quantile of the distribution, or the maximum observed displacement distance, whichever was smaller.
(DOCX)
