# Supplementary material for: Multi-level, multi-scale resource selection functions and resistance surfaces for conservation planning: Pumas as a case study
Source: PLoS One. 2017 Jun 13;12(6):e0179570. doi: 10.1371/journal.pone.0179570 (PMC5469479; doi:10.1371/journal.pone.0179570)
Supplement: S1 Table — We fit a Pareto distribution to the empirical distribution of displacement distances at each time-period and defined the maximum radii of the Pareto distribution by either using the 95th quantile of the distribution, or the maximum observed displacement distance, whichever was smaller. (DOCX) [file pone.0179570.s001.docx]

**S1 Table. Time-intervals and associated radii of Pareto kernels used to define available habitat for Point and Path Selection functions.**

We fit a Pareto distribution to the empirical distribution of displacement distances at each time-period and defined the maximum radii of the Pareto distribution by either using the 95^th^ quantile of the distribution, or the maximum observed displacement distance, whichever was smaller.

| **Time-**  **period (minutes)** | **Pareto scale** | **Pareto shape** | **Radius of Pareto kernel (meters)** |
| --- | --- | --- | --- |
|  |  |  |  |
| 5 | 9.26 | 0.85 | 241 |
| 20 | 18.87 | 0.78 | 408 |
| 40 | 37.88 | 0.71 | 681 |
| 60 | 57.88 | 0.66 | 915 |
| 80 | 73.42 | 0.65 | 1123 |
| 100 | 89.69 | 0.63 | 1317 |
| 120 | 124.12 | 0.58 | 1602 |
| 140 | 153.89 | 0.55 | 1850 |
| 160 | 197.16 | 0.49 | 2049 |
| 180 | 220.85 | 0.49 | 2298 |
| 200 | 249.70 | 0.44 | 2312 |
| 220 | 269.10 | 0.49 | 2797 |
| 240 | 316.75 | 0.47 | 3044 |
| 260 | 352.70 | 0.37 | 3104 |
| 280 | 394.34 | 0.42 | 3479 |
| 300 | 413.09 | 0.44 | 3819 |
| 320 | 429.02 | 0.49 | 3994 |
| 340 | 422.24 | 0.45 | 4099 |
| 360 | 453.27 | 0.43 | 4461 |
